# Identification of differentially expressed miRNAs in chicken lung and trachea with avian influenza virus infection by a deep sequencing approach

**DOI:** 10.1186/1471-2164-10-512

**Published:** 2009-11-05

**Authors:** Ying Wang, Vinayak Brahmakshatriya, Huifeng Zhu, Blanca Lupiani, Sanjay M Reddy, Byung-Jun Yoon, Preethi H Gunaratne, Jong Hwan Kim, Rui Chen, Junjun Wang, Huaijun Zhou

**Affiliations:** 1 Department of Poultry Science, Texas A&M University College Station, TX 77843-2472, USA; 2 Department of Biology & Biochemistry, University of Houston, Houston, TX 77204, USA; 3 Department of Veterinary Pathobiology, College of Veterinary Medicine, Texas A&M University, College Station, TX 77843-4467, USA; 4 Department of Electrical and Computer Engineering, Texas A&M University College Station, TX 77840, USA; 5 Department of Molecular and Human Genetics, Baylor College of Medicine, Houston 77030, TX, USA; 6 State Key Laboratory of Animal Nutrition, China Agricultural University, Beijing, 100193, PR China

## Abstract

**Background:**

MicroRNAs (miRNAs) play critical roles in a wide spectrum of biological processes and have been shown to be important effectors in the intricate host-pathogen interaction networks. Avian influenza virus (AIV) not only causes significant economic losses in poultry production, but also is of great concern to human health. The objective of this study was to identify miRNAs associated with AIV infections in chickens.

**Results:**

Total RNAs were isolated from lung and trachea of low pathogenic H5N3 infected and non-infected SPF chickens at 4 days post-infection. A total of 278,398 and 340,726 reads were obtained from lung and trachea, respectively. And 377 miRNAs were detected in lungs and 149 in tracheae from a total of 474 distinct chicken miRNAs available at the miRBase, respectively. Seventy-three and thirty-six miRNAs were differentially expressed between infected and non-infected chickens in lungs and tracheae, respectively. There were more miRNAs highly expressed in non-infected tissues than in infected tissues. Interestingly, some of these differentially expressed miRNAs, including miR-146, have been previously reported to be associated with immune-related signal pathways in mammals.

**Conclusion:**

To our knowledge, this is the first study on miRNA gene expression in AIV infected chickens using a deep sequencing approach. During AIV infection, many host miRNAs were differentially regulated, supporting the hypothesis that certain miRNAs might be essential in the host-pathogen interactions. Elucidation of the mechanism of these miRNAs on the regulation of host-AIV interaction will lead to the development of new control strategies to prevent or treat AIV infections in poultry.

## Background

Avian influenza virus (AIV) is a type A virus of the family *Orthomyxovirida*. Although wild aquatic birds such as water fowls and sea gulls are their natural reservoir [1], land-based birds including chickens may also be infected, which cause significant economic losses to the poultry industry, and raise a great public health threat due to potential host jump from animals to humans [2].

miRNAs are non-coding, single-stranded RNAs of 19~23 nucleotides which represent a novel class of gene regulators and play important roles in a variety of biological processes in both plants and animals [3-5]. miRNAs modulate gene expression largely at the post-transcriptional level by different mechanisms including direct cleavage of targeted mRNAs [4], inhibition of translation [6] or even up-regulation of translation [7]. miRNAs are involved in different biological activities such as development, differentiation, growth and metabolism [8-11]. Recently, in mammals, miRNAs have been reported to participate in the regulation of immunity, including development and differentiation of lymphocytes, monocytes and neutrophils, and modulation of inflammation [11]. MiR-150 expresses in mature B and T cells derived from mouse hematopoietic stem cells, and is able to block early B cell development when expressed prematurely [12]. MiR-181a is an intrinsic modulator of T cell sensitivity and selection in mice [13]. After exposure of THP-1 (human acute monocytic leukemia cell line) cells to lipopolysaccharides (LPS), miR-146 was identified as an inhibitor of signalling proteins of the innate immune responses by NF-kappaB [14]. miRNAs have also been found to be critical effectors in the regulation of viral pathogenesis. Two human encoded miRNAs (miR-136 and miR-507), have been shown to have potential binding sites for the genes that code for the polymerase basic 2 (PB2) and hemmagglutinin (HA) proteins and are reported to be involved in the pathogenesis of H5N1 AIV [15]. All of these evidences suggest that certain miRNAs might be important in the modulation of AIV infections in chickens.

In order to effectively control AIV infection in poultry, it is essential to elucidate the mechanisms of virus pathogenesis in chickens. However, how host cells interact with AIVs during infection in poultry remains poorly understood. Identification of differentially expressed miRNAs in AIV infected chickens will pave a novel avenue to understand host-virus interaction. With the development of next generation sequencing, massively parallel sequencing holds great promise for expression profiling [16] and it can provide a superior sensitivity at high sequencing depth to discover especially those miRNAs with low abundance and novel miRNAs that are not able to be identified using traditional cloning approaches. Deep sequencing has been previously used to profile both chicken miRNAs and Marek's disease virus miRNAs [17,18]. In the current study, a Solexa Sequencer was used to deep sequence differentially regulated chicken miRNAs in H5N3 infected and non-infected SPF chickens. Our results will expand the list of miRNAs which might be related to the host immune responses in animals.

## Results

### Virus titration

Virus replication was examined by real-time RT-PCR for influenza matrix gene from total RNAs of lung and tracheae at 4 dpi. The titer of infected samples was 12.29 log_10 _EID_50_/ml in lung, and 3.89 log_10 _EID_50_/ml in tracheae. Both non-infected lung and tracheae samples were negative.

### Small RNA libraries

**Figure 1:**
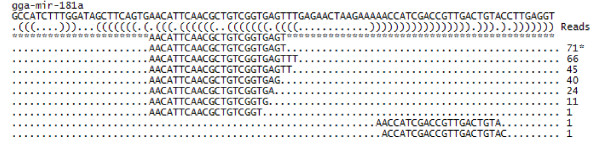
**Reads aligning with of gga-mir-181a**. Sequence of gga-mir-181a hairpin is described in the top line. The brackets below denote the secondary structure. Reads aligning with the mature gga-mir-181a sequence as found in miRBase is denoted by a '*' sequence. The +/- 4 nt matches representing reads that perfectly matched the gga-mir-181a precursor with insertions or deletion of 1-4 nucleotides from the 5' and 3' ends are shown below the mature miRNA exact matches. The number of reads corresponding to each sequence is shown at the right hand end of the dotted line.

A total of 278,398 and 340,726 filtered high quality reads were obtained from chicken lungs and tracheae, respectively (Table 1). In the libraries of chicken lungs, 98,849 and 179,549 reads were obtained from infected and non-infected lungs, respectively. Out of these reads, 52,363 of these high quality reads were exact matches while another 9,357 reads were loose matches to known chicken miRNAs. All reads with a perfect match to mature miRNA sequences from chicken deposited in miRBase  with insertions or deletions of 1-4 nucleotides was considered as a loose match to represent dicer-processing products from each of the chicken miRNA precursors. An example from gga-mir-181a is shown in Figure 1. Here we saw that the sample had 77 copies of a sequence that was identical to that of the mature gga-mir-181a (denoted by a '*'). In addition we observed significant alternate processing at the 3'-end that was characteristic of miRNAs in various copy numbers. Loose matches were defined by sequence reads that aligned with chicken miRNA consensus sequence with 1-4 mismatches. These may represent sequencing errors (when occurring in low copy numbers), mutations and/or RNA editing events. In the libraries of chicken trachea, 250,460 reads were obtained from infected tracheae and 90,266 were obtained from non-infected tracheae. Out of these reads, 44,243 of these high quality reads were exact matches and another 4,178 reads were loose matches to known chicken miRNAs. The sum of exact and loose match reads was used as the total number of reads for each miRNA.

**Table 1:** Number of reads of miRNAs from AIV infected and non-infected chicken lungs and tracheae

	***Infected lungs***	***Non-infected lungs***	***Infected tracheae***	***Non-infected tracheae***
High quality/both adapter	98,849	179,549	250,460	90,266
Exact match to known chicken miRNAs	10,939	41,424	36,405	7,838
Loose match to known chicken miRNAs	1,441	7,916	2,926	1,252

**Figure 2:**
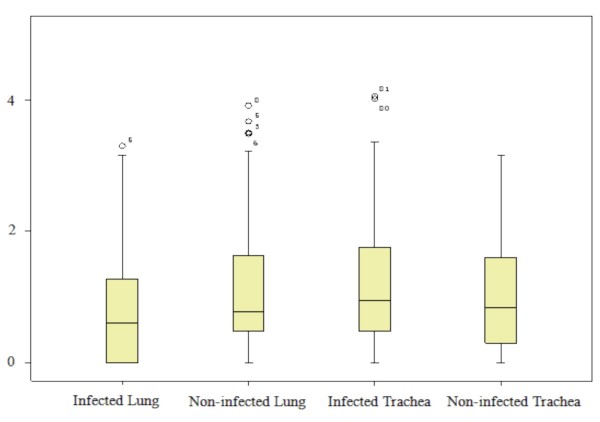
**Distributions of reads from each group**. Notes: All the reads in each group have been transformed by Log_10_.

To display the distribution of miRNAs at each library, reads of individual miRNA within each group were transformed to log_10_. The plots of distributions of transformed reads for each miRNA from each group are shown in Figure 2. Most miRNAs had around 10-100 reads in these four groups. There was no significant difference in the medians of the four libraries (P > 0.05) indicating they had similar distributions of the miRNA reads.

Of the 474 distinct *Gallus gallus *(gga) miRNA entries in miRBase [19-22], 377 miRNAs were identified in chicken lungs and 149 miRNAs in chicken tracheae. In addition, we identified 87 potential novel miRNA sequences [see Additional file 1]. Seven novel miRNAs were selected and all of them were confirmed by Real-time quantification PCR in chicken lungs and tracheae, respectively, which is shown in Table 2.

**Table 2:** Expression levels^1 ^of novel miRNAs in chicken lungs and tracheae.

	***Infected lungs***	***Non-infected lungs***	***Infected tracheae***	***Non-infected tracheae***
C1N1	312,681.30	127,134.80	83,221.38	260,694.40
C1N2	8,248,307.00	850,528.50	275,049.80	173,791.70
C1N3	7,546,275.00	966,448.50	726,699.80	3707461.00
C1N4	0.88	0.95	0.67	2.84
C1N5	395.08	263.26	58.67	329.24
C1N6	2,834.08	2,191.45	2,543.03	14,542.62
C1N7	121.38	31.89	172.25	1,088.66

Note: ^1 ^Expression levels were measured in terms of threshold cycle value (CT) and normalized to U6 using 2^-ΔΔCT^.

### miRNAs expression profiling analysis

In the present study there were four different groups: two tissues (lung and trachea) and two states per tissue (infected and non-infected states). Two comparisons were made between infected *vs*. non-infected within tissue and another two comparisons between lung and trachea at either infected or non-infected state. Differentially expressed miRNAs in each comparison were identified (P < 0.05, Q < 0.05 and fold change > 2). Within tissues, 73 and 36 miRNAs were differentially expressed between infected and non-infected groups in lungs and tracheae, respectively (Tables 3, 4). When between tissues were compared, 57 and 78 miRNAs were differentially expressed at infected and non-infected states, respectively (Tables 5, 6).

**Table 3:** Differentially expressed miRNAs between infected and non-infected lungs (P < 0.05, Q < 0.05 and Ratio > 2)

***miRNA***	***Position on chromosomes***	***Reads in infected***	***Reads in non-infected***	***Ratio infected/non*** ***-infected (Normalized)***
gga-mir-1576	chr13:18532632-18532726	8	0	-^1^
gga-mir-1636	chr 15: 4729959-4730046	6	0	-
gga-mir-206	chr3: 110390439-110390514	101	9	20.38
gga-mir-1793	chr9: 25115521-25115617	25	5	8.36
gga-mir-1599	chr7: 25926968-25927029	48	13	6.71
gga-mir-1701	chr4: 82234261-82234337	27	9	5.45
gga-mir-449	chrZ: 16040613-16040698	38	14	4.93
gga-miR-140	chr11: 21030641-21030735	1438	803	3.25
gga-mir-1416	chrZ: 34596479-34596567	17	10	3.09
gga-mir-1458	chr9: 11743528-11743637	25	15	3.03
gga-mir-1612	chr9: 6031748-6031831	39	31	2.29
gga-mir-1a-1	chr20: 8107831- 8107901	551	471	2.12
gga-mir-1a-2	chr2: 105673483- 105673567	593	523	2.06
gga-mir-103-2	chr4: 91906889-91906971	49	148	0.49
gga-mir-99a	chr1: 102424333-102424413	23	92	0.45
gga-mir-456	chr3: 32679710-32679821	30	122	0.45
gga-let-7c	chr1: 102425086-102425169	735	3059	0.44
gga-mir-181b-2	chr17: 10220137- 10220221	20	89	0.41
gga-let-7j	chr26: 1442697-1442779	648	2938	0.40
gga-mir-15b	chr9: 23742966- 23743056	9	41	0.40
gga-let-7a-1	chr12: 6302911-6303000	658	3020	0.40
gga-let-7a-2	chr24: 3380993-3381064	671	3113	0.39
gga-let-7g	chr12: 2809078-2809160	342	1623	0.38
gga-mir-181a-2	chr17: 10218497-10218587	57	283	0.37
gga-mir-200b	chr21: 2585642-2585726	14	72	0.35
gga-let-7a-3	chr1: 73421272-73421347	892	4699	0.34
gga-mir-100	chr21: 3372894-3372973	15	79	0.34
gga-mir-30d	chr2: 148337263-148337326	60	334	0.33
gga-mir-30e	chr23: 5248414-5248509	20	112	0.32
gga-mir-181a-1	chr8: 2001561-2001664	65	366	0.32
gga-mir-181b-1	chr8: 2001750-2001838	18	106	0.31
gga-let-7k	chr26: 1442897-1442979	53	327	0.29
gga-mir-125b	chr1: 102457647-102457736	19	119	0.29
gga-mir-1b	chr23: 4663912-4663975	10	63	0.29
gga-mir-146b	chr6: 24570060-24570164	102	691	0.27
gga-mir-10a	chrun_random: 379304-379377	86	598	0.26
gga-mir-101-2	chr8: 29051918-29051993	18	136	0.24
gga-mir-27b	chrZ: 41157642-41157738	5	41	0.22
gga-mir-144	chr19: 5824123-5824207	11	94	0.21
gga-let-7f	chr12: 6302497-6302583	947	8228	0.21
gga-mir-33-1	chr1: 51372282-51372350	2	19	0.19
gga-mir-126	chr17: 8431742-8431825	25	250	0.18
gga-mir-1456	chrZ: 44167486-44167595	2	20	0.18
gga-mir-22	chr19: 5352096- 5352195	4	43	0.17
gga-mir-16c	chr4: 4048689- 4048759	6	65	0.17
gga-mir-30a-3p	chr3: 85102239- 85102310	58	656	0.16
gga-mir-101	chrZ: 28037874- 28037952	19	219	0.16
gga-mir-30a-5p	chr3: 85102239- 85102310	68	785	0.16
gga-mir-30c-1	chr23: 5249637-5249725	3	37	0.15
gga-mir-146c	chr4: 92169271- 92169399	18	243	0.13
gga-mir-26a	chr2: 4467516- 4467592	98	1326	0.13
gga-mir-451	chr19: 5823968-5824036	93	1287	0.13
gga-mir-146a	chr13: 7555593- 7555691	7	105	0.12
gga-mir-21	chr19: 7322072-7322168	46	771	0.11
gga-mir-24	chrZ: 41158175-41158242	18	344	0.10
gga-mir-17-3p	chr1: 152248781-152248865	2	44	0.08
gga-mir-20a	chr1: 152248306-152248403	1	23	0.08
gga-mir-23b	chrZ: 41157406-41157491	12	285	0.08
gga-mir-142-3p	chr19: 496983-497070	2	49	0.07
gga-mir-142-5p	chr19: 496983-497070	2	49	0.07
gga-mir-17-5p	chr1: 152248781-152248865	2	55	0.07
gga-mir-19b	chr1: 152248183-152248269	1	31	0.06
gga-mir-30c-2	chr3: 85126853-85126924	1	34	0.05
gga-mir-739		7	241	0.05
gga-mir-15c	chr4: 4049055-4049130	1	46	0.04
gga-mir-15a	chr1: 173700493-173700575	2	102	0.04
gga-mir-16-2	chr9: 23742791-23742884	1	67	0.03
gga-mir-16-1	chr1: 173700351-173700434	1	107	0.02
gga-mir-106	chr4: 3970359-3970439	0	27	+^2^
gga-mir-1729	chr15: 769596-769666	0	24	+
gga-mir-1798	chr20: 9654914-9655009	0	24	+
gga-mir-429	chr21: 2580812-2580895	0	22	+
gga-mir-1711	chr12: 17010140-17010207	0	18	+

Note: ^1 ^Specifically expressed in infected lungs.

^2 ^Specifically expressed in non-infected lungs.

**Table 4:** Differentially expressed miRNAs between infected and non-infected tracheae (P < 0.05, Q < 0.05 and Ratio > 2)

***miRNA***	***Position on chromosomes***	***Reads in infected***	***Reads in non-infected***	***Ratio infected/non-infected (Normalized)***
gga-mir-1a-2	chr2: 105673483- 10567356	11423	441	9.34
gga-mir-1a-1	chr20: 8107831- 8107901	10438	405	9.29
gga-mir-455-3p	chr17:6021890-6021975	29	2	5.23
gga-mir-455-5p	chr17:6021890-6021975	29	2	5.23
gga-mir-34b	chr24: 5684900-5684983	870	82	3.82
gga-mir-499	chr20: 2599334-2599424	154	15	3.70
gga-mir-34c	chr24: 5685637-5685710	540	64	3.04
gga-mir-140	chr11: 21030641-21030735	2334	383	2.20
gga-mir-1b	chr23: 4663912-4663975	66	11	2.16
gga-mir-456	chr3: 32679710-32679821	37	28	0.48
gga-mir-125b	chr1: 102457647-102457736	31	24	0.47
gga-mir-148a	chr2: 32053543-32053610	116	94	0.44
gga-let-7b	chr1: 73422101-73422185	1761	1460	0.43
gga-mir-181a-1	chr8: 2001561-2001664	59	49	0.43
gga-mir-146c	chr4: 92169271- 92169399	42	36	0.42
gga-mir-181a-2	chr17: 10218497-10218587	51	45	0.41
gga-let-7g	chr12: 2809078-2809160	413	373	0.40
gga-mir-206	chr3: 110390439-110390514	95	88	0.39
gga-mir-222	chr1: 114216027-114216124; chr1: 114218422-114218519	14	13	0.39
gga-let-7k	chr26: 1442897-1442979	58	54	0.39
gga-mir-181b-2	chr17: 10220137- 10220221	20	21	0.34
gga-let-7i	chr1: 34895687-34895770	123	136	0.33
gga-mir-126	chr17: 8431742-8431825	17	19	0.32
gga-mir-99a	chr1: 102424333-102424413	14	16	0.32
gga-mir-30a-3p	chr3: 85102239- 85102310	66	76	0.31
gga-mir-30a-5p	chr3: 85102239- 85102310	74	87	0.31
gga-mir-146b	chr6: 24570060-24570164	78	107	0.26
gga-mir-181b-1	chr8: 2001750-2001838	18	25	0.26
gga-mir-30d	chr2: 148337263-148337326	46	68	0.24
gga-mir-100	chr21: 3372894-3372973	25	41	0.22
gga-mir-92	chr1: 152248070-152248070	24	40	0.22
gga-mir-15a	chr1: 173700493-173700575	3	9	0.12
gga-mir-451	chr19: 5823968-5824036	14	89	0.06
gga-mir-10a	chrun_random: 379304-379377	11	139	0.03
gga-mir-1612	chr9: 6031748-6031831	0	9	-^1^
gga-mir-1458	chr9: 11743528-11743637	0	7	-

Note: ^1 ^Specifically expressed in non-infected tracheae.

**Table 5:** Differentially expressed miRNAs between infected lungs and tracheae (P < 0.05, Q < 0.05 and Ratio > 2)

***miRNA***	***Position on chromosomes***	***Reads in lungs***	***Reads in tracheae***	***Ratio lung/trachea (Normalized)***
gga-mir-1599	chr7: 25926968-25927029	48	0	-^1^
gga-mir-1612	chr9: 6031748-6031831	39	0	-
gga-mir-1701	chr4: 82234261-82234337	27	0	-
gga-mir-1458	chr9: 11743528-11743637	25	0	-
gga-mir-1793	chr9: 25115521-25115617	23	0	-
gga-mir-181b-2	chr17: 10220137- 10220221	20	0	-
gga-mir-1416	chrZ: 34596479-34596567	17	0	-
gga-mir-7-1	chrZ: 8107831-8107901	16	0	-
gga-mir-7b	chr1: 73422101-73422185	15	0	-
gga-mir-1638	chr5: 58712377- 58712463	13	0	-
gga-mir-144	chr19: 5824123-5824207	11	0	-
gga-mir-1761	chr8: 17523212-17523292	9	0	-
gga-mir-1576	chr13:18532632-18532726	8	0	-
gga-mir-1814	chr4: 61722590-61722663	8	0	-
gga-mir-1452	chrZ: 8107831-8107901	7	0	-
gga-mir-1815	chr6: 29566734-29566810	7	0	-
gga-mir-122-1	chrZ: 649337-649413	6	0	-
gga-mir-122-2	chrurn_random: 12066796-12066872	6	0	-
gga-mir-1636	chr15: 4729959-4730046	6	0	-
gga-mir-1659	chr7: 14764187-14764287	6	0	-
gga-mir-1786	chr14: 7801714-7801822	6	0	-
gga-mir-218-1	chr4: 77774698-77774806	5	0	-
gga-mir-218-3		5	0	-
gga-mir-7-2	chr10: 14823525-14823623	5	0	-
gga-mir-1467	chr2: 141373919-141374028	4	0	-
gga-mir-1630	chr9: 1883593-1883649	4	0	-
gga-mir-1816	chr2: 90603851-90603955	4	0	-
gga-mir-7-3	chr28: 4436025-4436119	4	0	-
gga-mir-10a	chrun_random: 379304-379377	86	11	19.81
gga-mir-451	chr19: 5823968-5824036	93	14	16.83
gga-mir-184	chr10: 22146245-22146318	11	3	9.29
gga-mir-193b	chr14: 759453-759535	11	3	9.29
gga-mir-181a-2	chr17: 10218497-10218587	57	18	8.02
gga-mir-205a	chr26: 2896047-2896142	6	2	7.60
gga-mir-92	chr1: 152248070-152248070	42	24	4.43
gga-mir-99a	chr1: 102424333-102424413	23	14	4.16
gga-mir-126	chr17: 8431742-8431825	25	17	3.73
gga-let-7i	chr1: 34895687-34895770	177	123	3.65
gga-mir-146b	chr6: 24570060-24570164	102	78	3.31
gga-mir-30d	chr2: 148337263-148337326	60	46	3.30
gga-mir-181a-1	chr8: 2001561-2001664	65	51	3.23
gga-let-7b	chr1: 73422101-73422185	2008	1761	2.89
gga-mir-206	chr3: 110390439-110390514	101	95	2.69
gga-mir-148a	chr2: 32053543-32053610	114	116	2.49
gga-mir-30a-5p	chr3: 85102239- 85102310	68	74	2.33
gga-let-7k	chr26: 1442897-1442979	53	58	2.32
gga-mir-30a-3p	chr3: 85102239- 85102310	58	66	2.23
gga-mir-30e	chr23: 5248414-5248509	20	23	2.20
gga-let-7g	chr12: 2809078-2809160	342	413	2.10
gga-mir-456	chr3: 32679710-32679821	30	37	2.05
gga-mir-103-2	chr4: 91906889-91906971	65	82	2.01
gga-mir-1b	chr23: 4663912-4663975	10	66	0.38
gga-mir-34b	chr24: 5684900-5684983	112	870	0.37
gga-mir-34c	chr24: 5685637-5685710	78	540	0.37
gga-mir-499	chr20: 2599334-2599424	13	154	0.21
gga-mir-1a-1	chr20: 8107831- 8107901	551	10438	0.13
gga-mir-1a-2	chr2: 105673483- 10567356	593	11423	0.13

Note: ^1 ^Specifically expressed in infected lungs.

**Table 6:** Differentially expressed miRNAs between non-infected lungs and tracheae (P < 0.05, Q < 0.05 and Ratio > 2)

***miRNA***	***Position on chromosomes***	***Reads in lungs***	***Reads in tracheae***	***Ratio lung/trachea (Normalized)***
gga-mir-30c-2	chr3: 85126853-85126924	34	0	-^1^
gga-mir-19b	chr1: 152248183-152248269	31	0	-
gga-mir-1798	chr20: 9654914-9655009	24	0	-
gga-mir-1456	chrZ: 44167486-44167595	20	0	-
gga-mir-1711	chr12: 17010140-17010207	18	0	-
gga-mir-122-1	chrZ: 649337-649413	17	0	-
gga-mir-203	chr:5: 53206814-53206911	16	0	-
gga-mir-122-2	chrurn_random: 12066796-12066872	14	0	-
gga-mir-1599	chr7: 25926968-25927029	13	0	-
gga-mir-1638	chr5: 58712377- 58712463	12	0	-
gga-mir-1761	chr8: 17523212-17523292	12	0	-
gga-mir-144	chr19: 5824123-5824207	94	2	23.63
gga-mir-146a	chr13: 7555593- 7555691	105	3	17.60
gga-mir-739		241	7	17.31
gga-mir-106	chr4: 3970359-3970439	27	1	13.57
gga-mir-16-1	chr1: 173700351-173700434	107	4	13.45
gga-mir-193a	chr18: 6423770-6423846	26	1	13.07
gga-mir-142-3p	chr19: 496983-497070	49	2	12.32
gga-mir-142-5p	chr19: 496983-497070	49	2	12.32
gga-mir-1729	chr15: 769596-769666	24	1	12.07
gga-mir-20a	chr1: 152248306-152248403	23	1	11.56
gga-mir-21	chr19: 7322072-7322168	771	40	9.69
gga-mir-17-5p	chr1: 152248781-152248865	55	3	9.22
gga-mir-16-2	chr9: 23742791-23742884	67	4	8.42
gga-mir-24	chrZ: 41158175-41158242	344	21	8.24
gga-mir-30e	chr23: 5248414-5248509	112	7	8.04
gga-mir-15c	chr4: 4049055-4049130	46	3	7.71
gga-mir-223	chr:4: 232949-233048	15	1	7.54
gga-mir-29a	chr1: 3236329-3236417	15	1	7.54
gga-mir-29c	chr26: 2511658-2511746	15	1	7.54
gga-mir-17-3p	chr1: 152248781-152248865	55	3	7.37
gga-mir-451	chr19: 5823968-5824036	1287	89	7.27
gga-mir-101	chrZ: 28037874- 28037952	219	16	6.88
gga-mir-126	chr17: 8431742-8431825	250	19	6.61
gga-mir-26a	chr2: 4467516- 4467592	1326	103	6.47
gga-mir-130c	chr19: 7145027-7145120	25	2	6.28
gga-mir-23b	chrZ: 41157406-41157491	285	23	6.23
gga-mir-30c-1	chr23: 5249637-5249725	37	3	6.20
gga-mir-193b	chr14: 759453-759535	23	2	5.78
gga-mir-101-2	chr8: 29051918-29051993	136	12	5.70
gga-mir-15a	chr1: 173700493-173700575	102	9	5.70
gga-let-7f	chr12: 6302497-6302583	8228	781	5.30
gga-mir-27b	chrZ: 41157642-41157738	41	4	5.15
gga-mir-30a-5p	chr3: 85102239- 85102310	785	87	4.54
gga-mir-30a-3p	chr3: 85102239- 85102310	656	76	4.34
gga-mir-16c	chr4: 4048689- 4048759	65	8	4.08
gga-mir-181a-1	chr8: 2001561-2001664	366	49	3.76
gga-mir-200b	chr21: 2585642-2585726	72	10	3.62
gga-mir-22	chr19: 5352096- 5352195	43	6	3.60
gga-let-7a-3	chr1: 73421272-73421347	3193	468	3.43
gga-mir-146c	chr4: 92169271- 92169399	243	36	3.39
gga-mir-146b	chr6: 24570060-24570164	691	107	3.25
gga-mir-181a-2	chr17: 10218497-10218587	283	45	3.16
gga-let-7k	chr26: 1442897-1442979	327	54	3.04
gga-mir-15b	chr9: 23742966- 23743056	41	7	2.94
gga-let-7a-2	chr24: 3380993-3381064	3113	540	2.89
gga-mir-99a	chr1: 102424333-102424413	92	16	2.89
gga-mir-1b	chr23: 4663912-4663975	63	11	2.88
gga-mir-199-1	chr17: 5667150-5667243	541	95	2.86
gga-mir-199-2	chr8: 4732773-4732880	541	95	2.86
gga-let-7a-1	chr12: 6302911-6303000	3020	532	2.85
gga-let-7a-3	chr1: 73421272-73421347	1506	266	2.85
gga-let-7j	chr26: 1442697-1442779	2938	522	2.83
gga-mir-221	chr1: 114218926-114219024	129	23	2.82
gga-mir-128-1	chr7: 3222815032228231	43	8	2.70
gga-mir-125b	chr1: 102457647-102457736	119	24	2.49
gga-mir-30d	chr2: 148337263-148337326	344	68	2.47
gga-mir-103-2	chr4: 91906889-91906971	243	51	2.40
gga-let-7i	chr1: 34895687-34895770	633	136	2.34
gga-mir-107	chr6: 20487964-20488044	77	17	2.28
gga-let-7c	chr1: 102425086-102425169	3059	679	2.26
gga-mir-456	chr3: 32679710-32679821	122	28	2.19
gga-let-7g	chr12: 2809078-2809160	1623	373	2.19
gga-mir-10a	chrun_random: 379304-379377	598	139	2.16
gga-mir-181b-1	chr8: 2001750-2001838	106	25	2.13
gga-mir-181b-2	chr17: 10220137- 10220221	89	21	2.13
gga-mir-34b	chr24: 5684900-5684983	337	82	2.07
gga-mir-206	chr3: 110390439-110390514	9	88	0.05

Note: ^1 ^Specifically expressed in non-infected lungs.

More miRNAs (60 out of 73 miRNAs in lungs and 27 out of 36 miRNAs in tracheae) were down-regulated than up-regulated with AIV infection in both lungs and tracheae. When infected *vs*. non-infected was compared, 5 miRNAs (miR-106, miR-1729, miR-1798, miR-429 and miR-1711) were only expressed in the non-infected lungs, while 2 miRNAs (miR-1576 and miR-1636) were only expressed in infected lungs (Table 3). Between infected and non-infected tracheae, two (miR-1612 and miR-1458) out of 27 down-regulated miRNAs were expressed only in non-infected tracheae (Table 4).

In the comparisons between tissues, only few miRNAs (6 out of 57 miRNAs in the infected state and 1 out of 78 miRNAs in the non-infected state) were highly expressed in tracheae compared to lungs (Tables 5, 6). Under the infected state, 28 miRNAs were specifically expressed in lungs and 23 miRNAs were expressed at higher levels in lungs than in tracheae. In the non-infected state, 11 miRNAs specifically expressed in lungs and 66 miRNAs were expressed at higher levels in lungs than tracheae. Of particular interest, miR-1a, miR-140, and miR-449, which were highly expressed in infected tracheas than the non-infected ones, and also were differentially expressed between infected tissues (higher expression levels in infected tracheae than infected lungs). In the tissue comparison under the non-infected state, miR-206 was the only miRNA that had higher expression level in tracheae than in lungs. In general, those highly abundant miRNAs were observed across all four groups examined (Table 7).

**Table 7:** Highly abundant miRNAs in all four libraries

***Name***	***Uninfected lung*** ***(179,549)***^***1***^	***Infected lung*** ***(98,849)***	***Uninfected trachea*** ***(90,266)***	***Infected trachea*** ***(250,460)***
gga-let-7b	4,732	2,008	1,460	1,761
gga-let-7f	8,228	947	781	1,949
gga-let-7a-3	3,193	562	468	917
gga-let-7c	3,059	735	679	1,327
gga-mir-140	803	1,438	383	2,334
gga-mir-1a-2	523	593	441	11,423
gga-mir-1a-1	471	551	405	10,438
gga-let-7a-2	1,633	341	276	549
gga-let-7g	1,623	342	373	413
gga-let-7a-1	1,510	329	266	532

Note: ^1 ^Total number of reads for each group

### Confirmation of differentially expressed miRNAs

TaqMan miRNA assays were used to confirm the expression pattern of differentially expressed miRNAs in lungs. There were general consistency between TaqMan assay and deep sequence analysis in three miRNAs (miR-1a, miR-125b and miR-146a) in terms of directions of regulation and significance. Specifically, there was a 1.16 fold up-regulation (2.12 fold in deep sequencing analysis) in miR-1a, 2.13 fold down-regulation (8.33 fold in deep sequencing analysis) in miR-125b, and 3.03 fold down-regulation (3.45 fold in deep sequencing analysis) in miR-146a (P < 0.05).

### Clustering of chicken miRNAs

Chromosomal positions of differentially expressed miRNAs revealed that some of them were very close to each other. According to a previous report [23], miRNAs can be grouped as one cluster if they are less than 1,000 bp apart on the same chromosome. Based on the miRBase 13.0 [19-22], there are 20 miRNA clusters in the chicken genome according to the criteria above. Eighteen of these clusters were detected in lungs and 12 clusters in tracheae, respectively (Table 8). Each cluster contained at least two miRNAs, and total of 47 miRNAs were included in these clusters. Within these clusters, the mir-92-mir-19b-mir-20a-mir-19a-mir-18a-mir-17, which is equivalent to the mammalian mir-17-92 cluster, and mir-302b-mir-302c-mir-1811-mir-302a-mir-302d-mir-367 cluster were the biggest clusters containing six miRNAs. Both of them were detected in lungs. There were only seven clusters differentially expressed (all miRNAs within the cluster differentially expressed in the comparison of infection *vs*. non-infection or between tissues). Clusters mir-16-1-mir-15a, let-7f-let-7a-1, mir-181a-1-mir-181b-1, let-7j-let-7k, mir-23b-mir-27b-mir-24, and mir-16-2-mir-15b were down-regulated in lungs and mir-181a-1-mir-181b-1 was also down-regulated in tracheae with AIV infection. Cluster mir-34b-mir-34c was up-regulated in tracheae.

**Table 8:** Identified miRNA clusters

***Cluster***	***Chromosome locations***	***Expressed in lungs***	***Expressed in tracheae***
let-7a-3-let-7b	Chr1: 73421272-73421347; 73422101- 73422185	Y^1^	Y
mir-222-mir-221	Chr1: 114218422- 114218519; 11421 8926-114219024	Y	Y
mir-92-mir-19b-mir-20a-mir-19a-mir-18a-mir-17	Chr1: 152248070-152248147; 152248 183-152248269; 152248306- 1522484 03; 152248492 -152248572; 1522486 26-152248718; 152248781-152248865	Y	N^2^
mir-16-1-mir-15a	Chr1: 173700351-173700434; 173700 493-173700575	Y	Y
mir-20b-mir-18b	Chr4: 3970047-3970131; 3970228- 3970311	Y	N
mir-302b-mir-302c-mir-1811-mir-302a-mir-302d-mir-367	Chr4: 58651314-58651385; 58651576- 58651640; 58651698-58651778; 5865 1879-58651945; 58652214-58652282; 58652350-58652422	Y	N
mir-1547-mir-204-2	Chr10: 6651001-6651074; 6651274- 6651374	Y	N
mir-1720-mir-7-2	Chr10: 14823390-14823454; 14823525 -14823623	Y	Y
let-7f-let-7a-1	Chr12: 6302497-6302583; 6302911- 6303000	Y	Y
mir-1763-mir-1564	Chr14: 12895655-12895720; 12896507-12896577	Y	N
mir-34b-mir-34c	Chr24: 5684900-5684983; 5685637- 5685710	Y	Y
let-7j-let-7k	Chr26: 1442697-1442779; 1442897- 1442979	Y	Y
mir-29c-mir-29b-2	Chr26: 2511658-2511746; 2512569- 2512648	Y	N
mir-181a-1-mir-181b-1	Chr8: 2001561-2001664; 2001750- 2001838	Y	Y
mir-1b-mir-133c	Chr23: 4663912-4663975; 4664051- 4664129	N	Y
mir-449-mir-449b	ChrZ: 16040613-16040698; 16040763 -16040856	Y	Y
mir-216b-mir-1461	Chr3: 288214-288302; 288216-288301	N	N
mir-23b-mir-27b-mir-24	ChrZ: 41157406-41157491; 41157642- 41157738; 41158175-41158242	Y	Y
mir-194-mir-215	Chr3: 19924487-19924561; 19924793 -19924897	Y	N
mir-16-2-mir-15b	Chr9: 23742791-23742884; 23742966 -23743056	Y	Y

Note: ^1 ^Identified in the library

^2 ^Not identified in the library

### Gene ontology analysis

**Figure 3:**
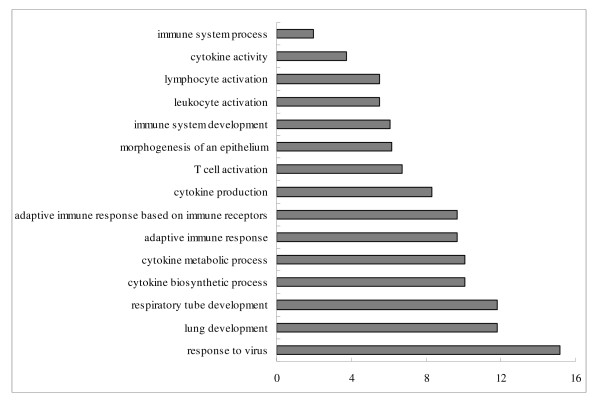
**Enriched immune related GO terms of target genes of repressed differentially expressed miRNAs in the comparison of infected *vs*. non-infected lungs**. Notes: Fold enrichment is a ratio obtained by dividing user's percentage by the percentage of each category of the whole genome.

**Figure 4:**
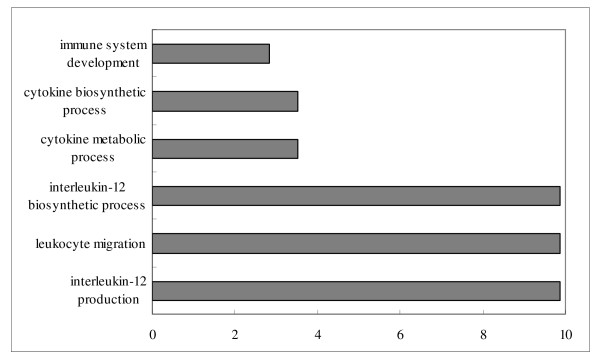
**Enriched immune related GO terms of target genes of repressed differentially expressed miRNAs in comparison of infected *vs*. non-infected tracheae**. Notes: Fold enrichment is a ratio obtained by dividing user's percentage by the percentage of each category of the whole genome.

**Figure 5:**
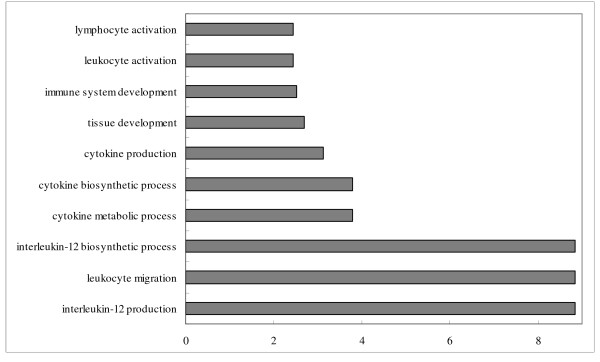
**Enriched immune related GO terms of target genes of induced differentially expressed miRNAs in comparison of infected lungs and tracheae**. Notes: Fold enrichment is a ratio obtained by dividing user's percentage by the percentage of each category of the whole genome.

**Figure 6:**
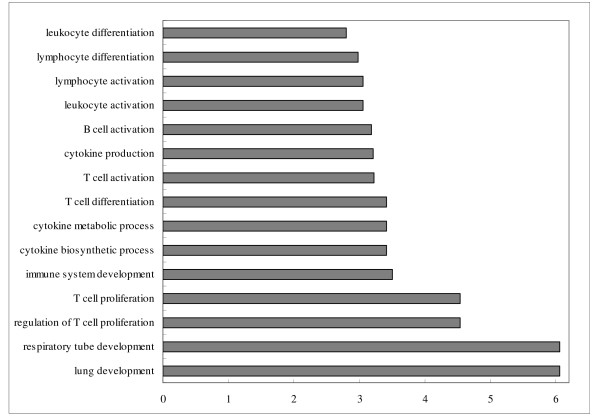
**Enriched immune related GO terms of target genes of induced differentially expressed miRNAs in comparison of non-infected lungs and tracheae**. Notes: Fold enrichment is a ratio obtained by dividing user's percentage by the percentage of each category of the whole genome.

Potential target genes of differentially expressed miRNAs in each comparison were predicted by miRanda [24]. In brief, each differentially expressed miRNA was submitted to miRanda individually and all of its targets predicted in miRanda were used for the following gene ontology (GO) analysis. For each comparison, target genes of induced and repressed miRNAs were separately analyzed. All targets of induced miRNAs in each comparison were submitted to DAVID program [25] and so were the targets of repressed miRNAs. Functional category enrichment based on the GO terms was evaluated on the targets of these differentially expressed miRNAs. All enriched GO terms in biological process of each comparison were shown in Additional file 2. Immune related GO terms of each comparison are presented in Figures 3, 4, 5 and 6.

In the comparison of infected *vs*. non-infected lungs, 15 immune related GO terms in biological process were significantly enriched (P < 0.05) (Figure 3) from the targets of down-regulated miRNAs. Response to virus was the GO term with the highest fold enrichment (15 folds). Other functional terms including immune system response, lymphocytes, and lung development were also identified.

In the comparison of infected *vs*. non-infected tracheae, six immune-related GO terms in biological process were significantly enriched (P < 0.05) (Figure 4) from the targets of down-regulated miRNAs, in which interleukin-12 production and interleukin-12 biosynthetic process had the highest fold enrichment (9.8 folds). For the targets of up-regulated miRNAs between the infected *vs*. non-infected both in lung and trachea comparisons, only two immune related GO terms: immunoglobulin I set and immunoglobulin subtype 2 in INTERPRO category were significantly enriched.

For the tissue comparison under the infected state, targets of induced miRNAs in lungs were associated with ten immune related GO terms in biological process including tissue development, interleukin-12, lymphocytes cytokines and immune system development (P < 0.05) (Figure 5). Targets of repressed miRNAs in lungs were associated with immune system development and immunoglobulin subtype 2 in the INTERPRO category. Under the non-infected state, 15 immune related terms were significantly enriched in biological process in the targets of miRNAs highly expressed in lungs. These GO terms were related to lung development and host immune system (Figure 6). The GO term NF-kappaB binding was also enriched in molecular function (P < 0.05).

## Discussion

The impact of miRNAs expression on the understanding of molecular mechanisms in gene regulations has been remarkable. Although thousands of small RNAs have been identified over the last decade, the challenge remains to fully identify all small nuclear RNAs, especially very low abundant ones and to determine their individual functions. The majority of known miRNAs have been identified through traditional cloning method, which is both time consuming and labor intensive. The advantages of next-generation sequencing technologies have provided an innovative tool to look into the genome with unprecedented depth of coverage. Solexa deep sequencing is one of these high throughput technologies, by which miRNAs can be detected in any organism without prior sequence or secondary structure information. This technology has been used in many species including human, mice and birds [17,18,26-28]. Expression of miRNAs varies in different developmental stages [29-31]. Chicken miRNAs identified in the present study provided novel information in the profiling of miRNAs not only in AIV infected chickens, but also the two tissues (lung and trachea) that have not been previously examined for miRNA profiling in chickens. To our knowledge, this is the first study to profile chicken miRNAs in AIV infected chickens by deep sequencing approach. There are 474 chicken miRNAs predicted in miRBase 13.0 [19-22]. The deep sequencing results in the current study experimentally confirmed 377 miRNAs in chicken lungs and 149 miRNAs in chicken tracheae, and the approach is more powerful than other conventional technologies previously used in birds [30]. The identification of these chicken miRNAs will be very useful in further investigating the functions and regulatory mechanisms of miRNAs in the chicken.

Growing evidence has suggested a relationship between differential miRNA expression and human diseases [32,33]. miRNAs can regulate many aspects of the immune response, including the development and differentiation of B and T cells, proliferation of monocytes and neutrophils, antibody switching and the release of inflammatory mediators by regulating basic component of host immune system [34-36]. MiR-155 has been reported by several groups to play important roles in both innate and adaptive immune responses in mammals [11,37,38]. MiR-155 deficient mice lacked the capability to generate defensive immune responses and to develop lymphocytes, especially B-cell, and antigen-presenting cell functions [39]. However, miR-155 showed very low abundance in both lungs and tracheae and no significant differential expression was observed in the present study. Over expression of miR-181a in mature mouse T cells can augment the sensitivity to peptide antigens, while suppressing miR-181a expression can reduce sensitivity and impair both positive and negative selection [13]. Selective expressions of miR-181a in the thymus and miR-223 in the bone marrow have been shown to be involved in the differentiation of pluripotent hematopoietic stem cells into the various blood cells lineages including B and T cells [40,41]. In the present study, miR-223 was not significantly regulated while miR-181a was down-regulated in both infected lungs and tracheae. In addition, miR-181a had a higher expression level in lungs than in tracheae under both infected and non-infected states. The expression levels of miR-181a, 181a* and 181b were investigated in LPS activated and CD40-lignad activated macrophages of chickens, respectively [42]. Only miR-181b was expressed in the macrophage cell line HD11 as well in the spleen adherent cells and that its expression increased after activation by LPS or CD40-ligand [42]. In the current study, miR-181b had same expression pattern with miR-181a in both lung and trachea comparisons. These results suggest that miR-181a and miR-181b may be strong miRNA candidates that regulate host response to AIV infection, and warrant further investigation of their targets and regulation mechanism in chickens.

Although the interaction between miRNA expression and virus infection remains to be elucidated, we speculated that miRNA might target immune related genes or modulate virus replication. Sequencing of chicken miRNAs in Marek's disease virus (MDV) infected and non-infected chicken embryo fibroblast (CEF) indicated that more miRNAs were up-regulated in MDV infected cells [17]. These results differ from the current study in which most differentially expressed miRNAs (55 out of 73 in lungs and 27 out of 36 in tracheae) were down-regulated in AIV infected tissues. These results indicate that the mechanisms of miRNA regulation of the host response to different types of virus in chickens are different. Chicken miR-221 and miR-222, the most abundant miRNAs in the CEF small RNA libraries, had significantly higher reads in MDV infected than non-infected CEF [17]. While both miR-221 and 222 had relatively lower abundance in the present study. These results demonstrate that miRNA expression can be tissue-specific with high abundance of miR-221 and 222 in the CEF libraries and low abundances in lungs and tracheae. It can also be speculated that host miRNAs expression may be suppressed by AIV replication based on the miRNA expression patterns observed in the current study.

Some miRNAs have been shown to be directly involved in virus replication. A liver specific miRNA (miR-122) was shown to be required for Hepatitis C virus (HCV) replication in humans [43]. MiR-122 can positively affect the viral replication and has become a therapeutic target for the treatment of HCV infection [44]. In the current study, miR-122 specifically expressed in chicken lungs compared to tracheae under both infected and non-infected states. These data suggest miR-122 might play a more important role in tissue distribution than the responses to AIV infection in chickens. Another two human miRNAs (miR-507 and miR-136) have potential target binding sites in polymerase basic 2 (PB2) and hemagglutinin (HA) genes of AIV, respectively [15]. Unfortunately, these two miRNAs are absent in the chicken genome, which might indicate different infectivity and lethality of the virus between chickens and humans.

Although in the present study most differentially expressed miRNA were down-regulated during AIV infection, some miRNAs were also up-regulated. MiR-1a, miR-140 and miR-449 were significantly up-regulated in both tissues, while miR-455, miR-34b and miR-34c were only up-regulated with AIV infection in tracheae. This suggests different miRNA regulation mechanisms might exist on host response to virus infection. These up-regulated miRNAs might inhibit gene expression of their target genes; therefore down-regulation of these target genes might help the host to inhibit virus replication.

Different tissues serve different biological functions in animals and the expression patterns of miRNAs can vary in different tissues [23,30]. miRNAs in bursa and spleen of developing chicken embryo have been recently identified, and diverse expression patterns of these miRNAs between different immune organs were observed, suggesting that miRNAs may function as dynamic regulators of the vertebrate immune system [45]. Some miRNAs show tissue-specific distribution in mouse, suggesting specific functions within these tissues [46]. In the current study, chicken lung and trachea were examined, as they are both part of the respiratory system and important sites for AIV replication. There was a significant difference in miRNA expression between lung and trachea with more miRNAs expressed in lungs (377 miRNAs identified) than tracheae (149 miRNAs identified), although only small percentage of miRNAs (19% in lung and 24% in trachea) were significantly differentially expressed in AIV infected samples.

When tissues in the state of virus infection were compared, 28 and 23 miRNAs were specifically and highly expressed in lungs, respectively, and only 6 miRNAs (miR-1a-1 and 2, miR-1b, miR-34b, 34c and miR-449) were highly expressed in tracheae (Table 5). When tissues were compared under the non-infected state, all differentially expressed miRNAs were expressed at higher levels in lungs than tracheae with the only exception of miR-206, which showed a higher expression level in non-infected trachea than lung (Table 6). More interestingly, miR-206 was up-regulated in virus infected *vs*. non-infected lungs and was down-regulated in infected *vs*. non-infected tracheae. We can conclude that miR-206 has an opposite regulatory role in lungs and tracheae or might have different targets in different tissues and therefore play different roles in host-virus interactions. MiR-1458 and miR-1612 were up-regulated in AIV infected chicken lungs, while they were specifically expressed in non-infected tracheae not the infected one (Tables 3 and 4). The different regulation of miR-1458 and miR-1612 between lung and trachea suggests they may also have different mechanisms in response to AIV infection between tissues.

We hypothesize that miR-34b, miR-34c, miR-206, miR-1458 and miR-1612 might be some of the most important miRNAs associated with AIV infection. Significantly different miRNA expression pattern between lung and trachea suggests the regulatory mechanism of miRNAs on host response to the AIV infection between lung and trachea is distinct. However, similar regulatory mechanism might also exist in these two tissues. Within the down-regulated miRNAs in infected *vs*. non-infected lungs and tracheae, there were 18 miRNAs which overlapped in both tissues. This suggests that these 18 miRNAs might have common modulation mechanisms with the AIV infection in chickens.

GO term enrichment analysis has been widely used in functional analysis and allows the identification of important categories associated with functions of interests. GO terms enriched by the target genes of differentially expressed miRNAs in the current study can provide useful information for the follow-up study to elucidate the regulatory mechanism of miRNAs in host immune response to AIV infection. During AIV infection, the host immune system is stimulated to develop a defensive mechanism, which might be the reason why genes involved in immune system development were enriched in all comparisons.

With virus infection, more immune related GO terms were enriched by the targets of repressed miRNAs in lungs than in tracheae (15 terms in lung comparison and 6 terms in trachea comparison) (Figures 3 and 4). Response to virus was identified as the most enriched term (15 fold enrichment) in lung comparison, confirming that genes related to virus infection were regulated by miRNAs. The hyperinduction of proinflammatory cytokines such as TNF-α and IFN-β in human macrophages and respiratory epithelial cells by the highly pathogenic AIV H5N1 was believed to contribute to its high pathogenecity [47]. Lymphocytes were also reported to be suppressed by AIV [48]. Enrichments of T-cell and leukocytes activation and cytokines activities terms identified in the comparison of infected *vs*. non-infected lungs might be an indication of host immune system response against virus infection. Meanwhile, GO terms involved in lung development and epithelium morphogenesis were enriched, suggesting the genes associated with lung epithelium development in lungs may be important for the recovery from AIV infection in chickens.

It was interesting that two GO terms, interleuklin-12 production and biosynthetic process, were enriched in the infected tissue comparison, which were not included in the non-infected comparison. These two terms were also enriched in the comparison of infected *vs*. non-infected trachea instead of the lung comparison. IL-12 plays a pivotal regulatory role in the anti-viral response due to its induction of IFN-γ, an anti-viral cytokine [49]. These may suggest that a different defensive mechanism against virus infection might occur in trachea compared to lungs.

The two terms, response to virus and T-cell activation were also enriched by immune related genes differentially expressed in the early immune responses to H9N2 infection in tracheal organ cultures (TOC) [50]. Host immune response, showed as adaptive immune responses in the current study, was enriched by differentially expressed genes in H5N1 infected chicken embryo fibroblasts (CEF) as well [51]. Influenza virus triggered a cascade of both innate and specific immune responses. Then both immune related genes and miRNAs who might regulate these genes maybe involved in similar biological processes with the same GO terms.

Of special note, NF-KappaB binding was also enriched in the comparison between lung and trachea under non-infected state. A similar GO term, regulation of NF-KappaB, was enriched in the previous TOC model with the infection of AIV H9N2 [50]. Activation of NF-KappaB pathway is an essential immediate early step of immune activation. Many viruses have developed strategies to manipulate NF-KappaB signalling through the use of multifunctional viral proteins that target the host innate immune response pathways [52]. Enrichment of GO term NF-KappaB binding suggests these two tissues might utilize this signal pathway differently.

Post-transcriptional gene activity can be regulated through the interaction of regulatory RNA-binding proteins and small non-coding RNAs such as miRNAs [4,53]. miRNAs can modulate protein activities by altering mRNA stability, translational efficiency or localization [53,54]. The 3' untranslated regions (3' UTR) are widely accepted as important post-transcriptional regulatory regions of mRNAs, which are particularly rich in *cis*-acting regulatory elements [55,56]. miRNAs can regulate their target genes through the *cis*-acting regulatory elements [57]. miRNAs within the same cluster might share the same cis-regulatory elements [23], and therefore, might have the same regulatory mechanism for their target genes. Out of the 18 miRNA clusters identified in lungs and 12 miRNA clusters identified in tracheae, there were 7 miRNA clusters differentially expressed in different comparisons. The miRNAs from five of these clusters (mir-16-1-mir-15a, mir-16-2-mir-15b, let-7f-let-7a-1, let-7j-let-7k and mir-23b-mir-27b-mir-24) identified in both lungs and tracheae were significantly down-regulated in infected lungs compared to non-infected lungs and also had higher expression levels in non-infected lungs than non-infected tracheae. The mir-181a-1-mir181b-1 cluster was significantly down-regulated in both infected lungs and tracheae. And the mir-34b-mir-34c cluster was the only significantly up-regulated cluster in the AIV infected trachea. Different miRNA clusters had different regulation direction in AIV infected tissues in the present study. This illustrates that, during AIV infection, different modulation mechanisms among different miRNA clusters might coexist in both lungs and tracheae.

It is interesting to note that when considering the miRNA clusters that were most active in chicken lung and trachea, mir-17-92 cluster (consisting of six miRNAs) and mir-302b-mir-302c-mir-1811-mir-302a-mir-302d-mir-367 cluster are highly associated with cell proliferation and self-renewal of stem cells and cancer cells [58-61]. In addition the miRNAs clusters that were significantly down-regulated miR-15/16 and let-7 are typically down-regulated in stem cells and cancer [62-64]. These results suggest that AIV infection in chickens may instigate cell proliferation and self-renewal like behaviour in chicken lung epithelium and the newly recruited T lymphocytes.

Modulation of target genes by miRNA is one of most critical steps for gene expression regulation. The targeted genes for some differentially expressed miRNAs in the current study were predicted using miRanda [24,65]. Interestingly, many of the target genes were involved in the host immune system. The potential target genes for miR-1a and miR-1b are the T-cell immuno-modulatory protein. MiR-34b and miR34c, whose target genes are B-cell CLL-pymphoma 2 & 11, might be involved in the B-cell differentiation. Target genes for miR-206 were associated with monocyte macrophage differentiation, suggesting they maybe associated with antigen presentation. Based on other immune related miRNA studies in mammals [11,66], differentially expressed miRNAs of their mammalian homologs and their targets are presented in Table 9. MiR-15a, miR-21 and miR-181a have important functions in lymphocytes development and modulations while miR-122 and miR-24 are related to virus infection and miR-146a, induced by macrophages, can activate Toll like receptor (TLR) and expose antigens to interleukin-1 beta. Although the exact functions of these miRNAs in the AIV infected chickens remains to be determined, candidate miRNAs and their potential targets identified in the current study provide strong evidence of their roles and warrant further investigation. Whether these chicken miRNAs have the same function as mammals or not need to be validated in the future studies. On-going efforts in the author's laboratory focusing on gene expressions of these target genes and determination of target genes for these differentially expressed miRNAs will provide new insights of miRNA regulations on AIV infection in chickens.

**Table 9:** miRNAs involvement in immune response [11]

***miRNA***	***Functions***	***Targets***
miR-15a	Decreased expression in chronic lymphocytic leukaemia	Bcl-2
miR-16	Binds to UA rich elements in 3' UTR and induces TNF alpha mRNA degradation	TNFα
miR-21	Increased expression in B-cell lymphoma and chronic lymphocytic leukaemia	
miR-17-5p	Inhibits monocyte proliferation, differentiation and maturation	AML-1
miR-20a	Inhibits monocyte proliferation, differentiation and maturation	AML-1
miR-106a	Inhibits monocyte proliferation, differentiation and maturation	AML-1
miR-24	Inhibits replication of vesicular stomatitis virus	
miR-29a	Down-regulated in B-cell chronic lymphocytic leukemia	Tcl-1
miR-122	Required for hepatitis C proliferation in liver	
miR-125b	Expression downregulated by LPS and oscillations in expression after exposure to TNF alpha	TNFα
miR-146a	Expression induced in macropahges and epithilial following activation of TLR or exposure to TNF alpha and IL-1beta	IRAK1, TRAF6
miR-146b	LPS induced expression induced in macrophages	IRAK1, TRAF6
let-7i	Regulates TLR-4 and contributes to cholangiocyte immune responses	
miR-181a	Positive regulator of B-cell development and CD4^+ ^T-cell selection, activation and sensitivity	SHP-2, PTPN22, DUSP5, DUSP6

## Conclusion

miRNAs have recently been implicated in the intricate cross-talk between host and pathogen in viral infections and are critical in viral pathogenesis. In AIV infected tissues, expression patterns of some host miRNAs were significantly differentially regulated in the current study, supporting the hypothesis that certain miRNAs are essential in the host-pathogen interactions. Once the role of these miRNAs in the regulation of host-AIV interaction has been determined, it will improve the protective strategies in AIV infection in poultry.

## Methods

### Sample collection and RNA isolation

One week old commercial Leghorn SPF chickens were randomly divided into two groups (4 chickens per group), housed in a negative pressure Horsfall-Bauer, temperature control isolation unit, and provided with water and commercial feed *ad libitum*. At three weeks of age, one group was inoculated with 0.2 ml H5N3 virus containing 106.0 EID_50_/ml, while the other group was inoculated with PBS by the intra-choanal cleft route. Based on the pilot study at 4 dpi, depression and severely congested lungs and trachea were observed. Therefore, all chickens were euthanized at four days post-inoculation, and lung and trachea epithelial layers were collected for RNA isolation. The animal experiment was performed according to the guidelines approved by the Institutional Animal Care and Use Committee, Texas A&M University.

Two pools of total RNA samples (two random chickens per pool) of each tissue from each group were generated. Total RNAs were isolated using Trizol (Invitrogen, Carlsbad, CA) following the manufacturer's protocol. Dnase I (Ambion, Austin, TX) digestion was carried out after RNA isolation according to manufacturer's instructions. The RNA concentration and purity were determined by measuring absorbance at 260 nm and A260/A280 ratio using a NanoDrop ND-1000 spectrophotometer (Nanodrop Technologies, Wilmington, DE). RNA samples were stored at -80°C until further use.

### Viral Titration

Virus replication at 4 dpi was determined by real-time RT-PCR for influenza matrix gene using AgPath-ID™ AIV- M kit (Ambion, Austin, TX) following the manufacturer's instructions. Control RNA was extracted from serially diluted H5N3 virus (10^1.5^-10^5.5 ^log_10 _EID_50_/ml). Standard curve was generated with control viral RNAs. The amount of RNA in the samples was converted into log_10 _EID50/ml by interpolation as described previously [67].

### Small RNA sequencing and analysis

For small RNA library construction, RNA samples were prepared using the DGE-Small RNA Sample Prep Kit (Illumina, San Diego, CA). In brief, RNA was purified by polyacrylamide gel electrophoresis (PAGE), to enrich for molecules in the range of 18-30 nt, and ligated with proprietary adapters to both 5' and 3' termini of the RNA. Ligated samples were used as templates for cDNA synthesis and then amplified with 15 PCR cycles to produce sequencing libraries. A total of eight Solexa-ready small RNA templates were prepared through two gel purification steps to eliminate concatenated adaptors without inserts. Purified cDNAs were quantified using the Quant-iT PicoGreen dsDNA Kit (Invitrogen, Carlsbad, CA) and diluted to 10 nM for sequencing on an Illumina 1G Genome Analyzer at the Genome Sequencing Center of Baylor College of Medicine. Cluster generation was performed and clusters were sequenced.

For each sample, sequences were first passed through an adaptor filter that searched for sequences that were followed by at least 6 nucleotides of the 3' adaptor. Out of the total reads, any reads without a perfect 10-nt linker subsequence were directly discarded adjoining the insert, yielding of length 10 nt or longer that were subject to further processing. All full-length, exact sequence matches to *E. coli *(k12, o157:h7, o157:h7 edl933, cft073) were discarded to eliminate possible sequence artifacts arising from the amplification process. All unique sequence reads with a minimum read count of 10 were aligned with precursor chicken miRNA sequences from miRBase version 13.0 [19-22]. Reads of each miRNA were the sum of exact and loose matches (± 4 bp) to known miRNAs. For each sample, counts were normalized to the total number of small RNA sequences, and then for each miRNA, the normalized number of counts was compared between groups or between tissues.

Fisher's Exact test was used to identify differentially expressed miRNAs at a 5% false discovery rate. False discovery rate (FDR) (Q values) was calculated by R program according to Benjamin and Hochberg's method [68]. Ratios were calculated as the ratio of normalized reads of infected over non-infected group or lung over trachea. Statistics related to over representation of functional categories were performed using DAVID, which is based upon a Fisher Exact statistic methodology similar to that described by Al-Shahrour et al [69]. A P < 0.05 was considered as significant.

Novel miRNAs from both lung and trachea libraries were identified using the method by Creighton et al. 2009 [70]. In brief, the first step is to take the sequence reads that did not map to known miRNA precursors, mapped them to the chicken genome, and got an exact sequence match along with 100 bases flanking either side. About 220-bp sequence was then tested for miRNA-like hairpin structure, and folded with the Vienna package [71]. The miRNA hairpin structures that meet the Ambros [22] criteria were identified. Specifically, the putative miRNA must lie on one arm of a single-loop hairpin with minimum free energy less than -25 kcal/mol. The sequence reads that were appropriately placed in these miRNA-like hairpins were considered as 'putative mature miRNAs' (pmms). Then we examined the pmms for cross-species conservation of the hairpin structure. The sequence reads with strong conservation of the mature miRNA, significant conservation of the hairpin arm opposite the mature miRNA, and little or no conservation of the hairpin loops were considered as novel miRNAs.

### Confirmation of novel miRNAs by miScript real-time quantitative RT-PCR

Novel potential miRNA expression was determined by using the same total RNA samples for small RNA library constructions. Total RNA (1 μg) was reverse-transcribed with miScript Reverse Transcription Kit from QIAGEN (Valencia, CA). The real-time quantification of a selected subset of novel miRNAs (C1N1: AAGCUGCCAGUUGAAGAACU; C1N2: AAGGUCCAACCUCACAUGUCC; C1N3: UUGGUGGUUCAGUGGUAGAA; C1N4: AGAAUUGCGUUUGGACAAUC; C1N5: CACAAGAAUUGCGUUUGGACAA; C1N6: UUGACAUCAUCAUACUUGGGAU and C1N7: UGGCAGUGCGUGUUAGCUGGCUGUU) was carried out with customer designed miScript Primer Assays and miScript SYBR Green PCR Kit from QIAGEN (Valencia, CA). Chicken small nuclear RNA U6 (GCAGGGGCCAUGCUAAUCUUCUCUGUAUCG) was used for normalization. The expression levels of novel miRNAs were measured in terms of threshold cycle value (CT) and normalized to U6 using 2^-ΔΔCT ^[72].

### Confirmation of differentially expressed miRNAs by TaqMan MicroRNA Assay

To determine the expression of miRNAs by quantitative RT-PCR (qRT-PCR), TaqMan microRNA assay was performed. The specific stem-loop RT primers of miR-1a, miR125b, miR-146a and U6 were obtained commercially from Applied Biosystems (Foster City, Calif., USA). In brief, cDNA was synthesized from total RNA by using the gene specific primers according to the protocol of TaqMan Micro RNA Assays (Applied Biosystems, CA, USA). Reverse transcriptase reactions contained 10 ng of RNA samples, 3 μl stem loop RT primer and reagents from a TaqMan MicroRNA Reverse Transcription Kit (Applied Biosystems, CA, USA). The 15 μl reactions were incubated for 30 min at 16°C, 30 min at 42°C and 5 min at 85°C, and then held at 4°C.

Real-time PCR was performed by using gene specific probes and a pair of primers (TaqMan MicroRNA Assays, Applied Biosystems) and reagents of TaqMan 2* Universal PCR Master Mix (No AmpErase UNG) (Applied Biosystems, CA, USA). The 20 μl PCR reactions included 1.33 μl RT-PCR product, 10 μl PCR master mix, and 1 μl 20* TaqMan MicroRNA Assay mix (Applied Biosystems, CA, USA). These reactions were incubated at 95°C for 10 min, followed by 40 cycles at 95°C for 10 s, 60°C for 40 s and 72°C for 1 s by ABI 7900 Realtime PCR instrument (Applied Biosystems, CA, USA). All reactions were run in triplicates. The threshold cycle was defined as the fractional cycle number at which the fluorescence passes the fixed threshold. The expression levels of miR-1a, miR-125b and miR-146a in each sample were measured in terms of threshold cycle value and normalized to U6 using 2^-ΔΔCT ^[72]. U6 was used as an internal control.

## Authors' contributions

YW carried out the RNA isolation, small RNA library construction preparation, analyzed data and drafted the manuscript. VB was responsible for the animal trial. BL and SR contribute to experiment design. BY, HFZ, JHK, JW, and PG contributed to the analysis of miRNA. RC ran the miRNA deep sequencing. HZ provided the concepts of the study, and revised the manuscript. All authors submitted comments, read and approved the final manuscript.

## Supplementary Material

Additional file 1 **Novel miRNAs**. This table includes all the novel miRNAs identified in both chicken lung and trachea in the current study. Click here for file

Additional file 2 **Enriched GO terms in biological process of each comparison**. This table includes all the enriched GO terms in biological process of the four comparisons. Click here for file
