# Correction: Identification of differentially expressed miRNAs in chicken lung and trachea with avian influenza virus infection by a deep sequencing approach

**DOI:** 10.1186/1471-2164-11-373

**Published:** 2010-06-11

**Authors:** Ying Wang, Vinayak Brahmakshatriya, Huifeng Zhu, Blanca Lupiani, Sanjay M Reddy, Byung-Jun Yoon, Preethi H Gunaratne, Jong Hwan Kim, Rui Chen, Ashley L Benham, Junjun Wang, Huaijun Zhou

**Affiliations:** 1 Department of Poultry Science, Texas A&M University College Station, TX 77843-2472, USA; 2 Department of Biology & Biochemistry, University of Houston, Houston, TX 77204, USA; 3 Department of Veterinary Pathobiology, College of Veterinary Medicine, Texas A&M University, College Station, TX 77843-4467, USA; 4 Department of Electrical and Computer Engineering, Texas A&M University College Station, TX 77840, USA; 5 Department of Molecular and Human Genetics, Baylor College of Medicine, Houston 77030, TX, USA; 6 State Key Laboratory of Animal Nutrition, China Agricultural University, Beijing, 100193, PR China

## Correction

After the publication of this work[1] we decided to submit all the novel microRNAs discovered from this study to miRBase as a part of this manuscript. All the novel miRNAs discovered have now been submitted to miRBase (http://www.mirbase.org/cgi-bin/mirna_summary.pl?org=gga) under the following miRBase IDs.

The novel microRNA discovery and analysis was performed by Ms. Ashley L. Benham. We therefore, would like to include her as an author of this manuscript. The corrected author list is below.

Ying Wang, Huifeng Zhu, Blanca Lupiani, Sanjay M Reddy, Byung-Jun Yoon, Preethi H Gunaratne, Jong Hwan Kim, Rui Chen, Junjun Wang and Huaijun Zhou have agreed to add Ms. Benham to the author list and communicated this through an email to your editorial office.

> miRBase communication: The following novel miRNAs have been submitted to

> > >gga-novel-mir-2132 gga-mir-2188

> > >gga-novel-mir-2133 gga-mir-3523

> > >gga-novel-mir-2134 tRNA

> > >gga-novel-mir-2135 gga-mir-3524

> > >gga-novel-mir-2143 gga-mir-3525

> > >gga-novel-mir-2136 gga-mir-3526

> > >gga-novel-mir-2137 gga-mir-3527

> > >gga-novel-mir-2138 gga-mir-3528

> > >gga-novel-mir-2139 gga-mir-3529

> > >gga-novel-mir-2140 gga-mir-3530

> > >gga-novel-mir-2141 gga-mir-2964

> > >gga-novel-mir-2142 gga-mir-3531

> > >gga-novel-mir-2144 gga-mir-3532

> > >gga-novel-mir-2145 gga-mir-3533

> > >gga-novel-mir-2146 gga-mir-3534

> > >gga-novel-mir-2147 gga-mir-3535

> > >gga-novel-mir-2148 gga-mir-3536

> > >gga-novel-mir-2149 gga-mir-3537

> > >gga-novel-mir-2150 gga-mir-3538-1

>

> > >gga-novel-mir-2151 gga-mir-3538-2

>

>

> > >gga-novel-mir-2152 gga-mir-3539

> > >gga-novel-mir-2153 gga-mir-3540

> >

> > Your novel-mir-2134 appears to be a tRNA fragment.

Correction: Identification of differentially expressed miRNAs in chicken lung and trachea with avian influenza virus infection by a deep sequencing approach

Ying Wang^1^, Vinayak Brahmakshatriya^1^, Huifeng Zhu^2^, Blanca Lupiani^3^, Sanjay M Reddy^3^, Byung-Jun Yoon^4 ^, Preethi H Gunaratne^2 ^, Jong Hwan Kim^2^, Ashley L. Benham^2^, Rui Chen^5^, Junjun Wang^6 ^and Huaijun Zhou^1^

1 Department of Poultry Science, Texas A&M University College Station, TX 77843-2472, USA

2 Department of Biology & Biochemistry, University of Houston, Houston, TX 77204, USA

3 Department of Veterinary Pathobiology, College of Veterinary Medicine, Texas A&M University, College Station, TX 77843-4467, USA

4 Department of Electrical and Computer Engineering, Texas A&M University College Station, TX 77840, USA

5 Department of Molecular and Human Genetics, Baylor College of Medicine, Houston 77030, TX, USA

6 State Key Laboratory of Animal Nutrition, China Agricultural University, Beijing, 100193, PR China
